# Experimentally constrained early reproduction shapes life history trajectories and behaviour

**DOI:** 10.1038/s41598-021-83703-1

**Published:** 2021-02-24

**Authors:** David Canal, Francisco Garcia-Gonzalez, László Zsolt Garamszegi

**Affiliations:** 1 Institute of Ecology and Botany, Centre for Ecological Research, Vácrátót, Hungary; 2 Center for the Study and Conservation of Birds of Prey in Argentina (CECARAUNLPam) & Institute of Earth and Environmental Science of La Pampa (INCITAP), National Scientific and Technical Research Council (CONICET), Santa Rosa, Argentina; 3 Estación Biológica de Doñana-CSIC, Seville, Spain; 4 Centre for Evolutionary Biology, School of Biological Sciences, University of Western Australia, Crawley, WA 6009 Australia; 5 MTA-ELTE, Theoretical Biology and Evolutionary Ecology Research Group, Department of Plant Systematics, Ecology and Theoretical Biology, Eötvös Loránd University, Budapest, Hungary

**Keywords:** Evolutionary biology, Behavioural ecology

## Abstract

The trade-off between current and future reproduction is a cornerstone of life history theory, but the role of within-individual plasticity on life history decisions and its connections with overall fitness and behaviour remains largely unknown. By manipulating available resources for oviposition at the beginning of the reproductive period, we experimentally constrained individual life history trajectories to take different routes in a laboratory study system, the beetle *Callosobruchus maculatus*, and investigated its causal effects on fecundity, survival and behaviour. Compared to females without resource limitations, females experiencing restricted conditions for oviposition had reduced fecundity early in life but increased fecundity when resources became plentiful (relative to both the previous phase and the control group) at the expense of longevity. Constrained reproduction in early life also affected behaviour, as movement activity changed differently in the two experimental groups. Experiencing reproductive constraints has, therefore, consequences for future reproduction investments and behaviour, which may lead individuals to follow different life history strategies.

## Introduction

A central tenet of life-history theory is the existence of trade-offs among fitness-related traits^1^. Trade-offs are ubiquitous, shape life history strategies and have profound consequences on trait evolution^2,3^. A key trade-off in life history concerns the investment between reproduction and survival. This trade-off typically implies a constraint between current and future reproduction, because, when resources are limited, a heavy investment in a given reproductive event may reduce resources for self-maintenance and thus, the chances to survive and reproduce in the future. Despite this clear-cut expectation, disentangling the causal factors underlying the resolution of these trade-offs is a major challenge in evolutionary biology^2,4,5^. Variation in resource acquisition and allocation may lead to positive (rather than negative) phenotypic correlations among individuals, thus masking the underlying trade-off^6^. Further, trade-offs may be contingent on particular environmental contexts, as they can elicit plasticity in the expression of traits and/or their correlations^2,5,7^.

Behavioural traits, which exhibit considerable within-individual variation, are often linked to fitness, but the extent to which trade-offs affect them remains elusive. In this regard, investment in somatic maintenance rather than reproduction under unfavourable conditions (and vice versa) may impact future behaviours related to mate and breeding resource acquisition (e.g. aggressiveness, exploration) or to offspring and own survival (e.g. feeding rate, risk taking), particularly in short-lived species in which survival expectancies decrease rapidly^1^. For instance, after experiencing suboptimal conditions, individuals might increase their dispersal tendency, explorative behaviour or movement activity to spread at larger distances where environmental conditions are favourable or competition is less intense^8^. Alternatively, individuals could be instead exhausted due to intense competition for resources, thus reducing their general levels of activity. Despite the importance of identifying causal relationships among life-history traits resulting from the trade-off between current and future reproduction, as well as their interplay with behavioural variation, most research focuses on phenotypic correlations, which preclude making straightforward inferences about how individual life history trajectories are shaped. Experimental manipulation is thus essential to understand the role of within-individual plasticity on life history decisions and its connections with overall fitness and behaviour.

**Figure 1:**
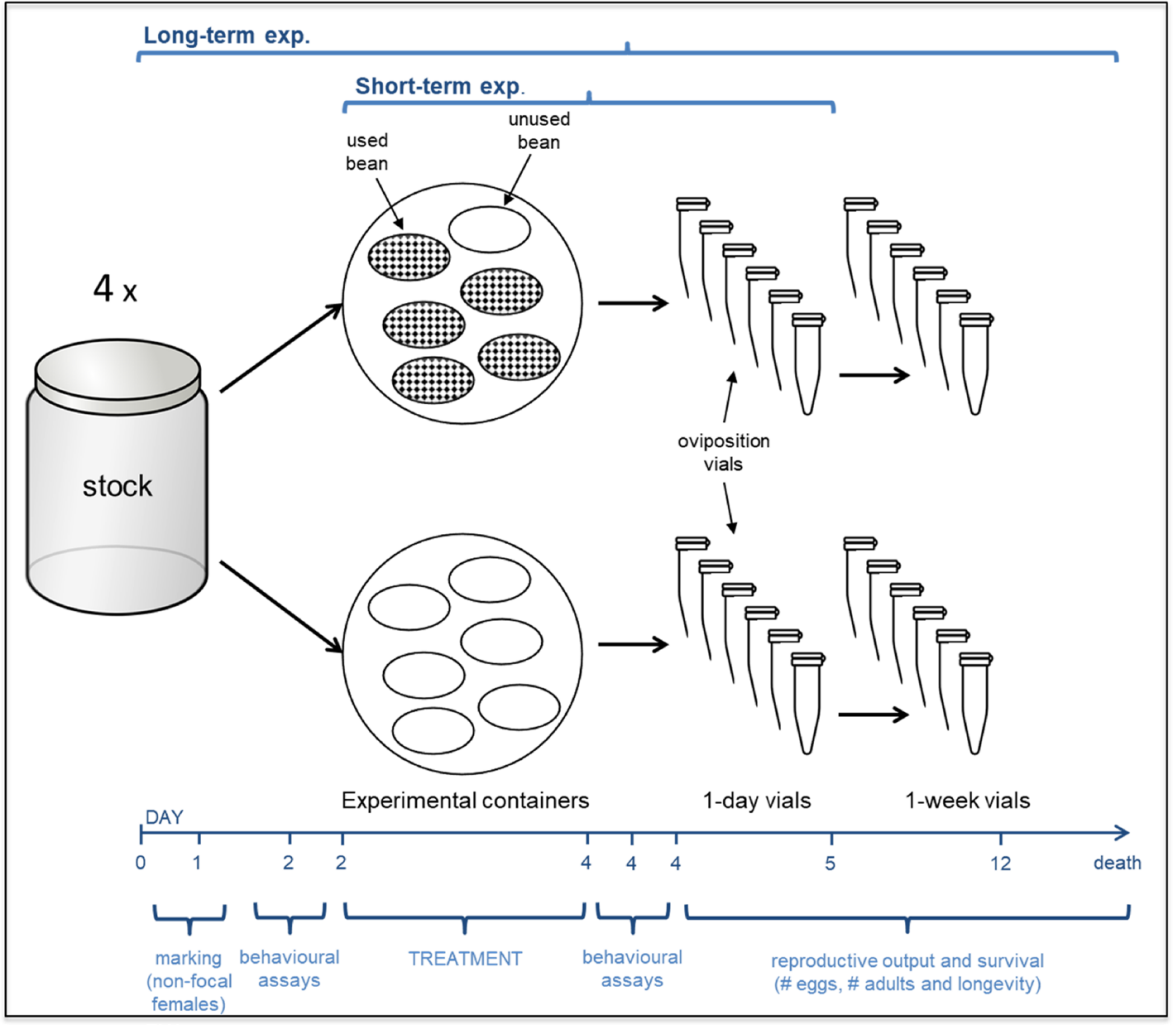
Schematic overview of the experimental setup. *Callosobruchus maculatus* females were exposed to either restricted or unrestricted resources for oviposition early in life. For simplicity only six beans are shown in each experimental container. In the long-term experiment we focused on long-term reproductive investments, as well as on behaviour and longevity. In a short-term experiment, we focused on reproductive investment during the treatment phase (as this could not be measured in the long-term experiment). The short-term experiment was a simplification of the long-term experiment: it ended on 1-day vial and no behavioural assays were conducted before and after the treatment. The experimental procedure of the long-term experiment was repeated four times (samples sizes per treatment for each experimental block are indicated in Table 1). For details of the protocols see main text.

Here, we used the seed beetle *Callosobruchus maculatus,* a capital breeder, to experimentally constrain reproductive investment early in life via limited resources for oviposition, whereby we induced competition for such resources. We assessed the effect of such manipulation on future reproductive effort, survival and movement activity. Movement activity has obvious consequences for fitness as it is a core component of key behaviours such as dispersal, predator avoidance or resource acquisition in numerous taxa^9,10^. Particularly, in *Callosobruchus* species, activity is positively associated with mating success, metabolic rates and it also modulates antipredator behaviours^11,12^. Activity, therefore, has profound repercussions on fitness through mate acquisition rates, acquisition of oviposition substrates and/or survival probabilities^12,13^. Our experiment (Fig. 1) relied on the manipulation of the amount of suitable substrate available for oviposition early in life. In particular, in a group of beetles we allowed individuals to mate and lay eggs under limited resources for oviposition to generate intense competition among laying females, while in another group, we provided plentiful resources, keeping the competition among laying females at a minimum. Females were assayed for behaviour before and after the experimental procedure and then placed, after the treatment, in vials under the same unlimited access to oviposition substrates to monitor their subsequent reproductive output throughout their life. Based on life-history theory, we formulated the following predictions. First, due to intense competition for oviposition resources, we expected restricted females to have lower reproductive investment and lower reproductive success (number of eggs laid and number of adult offspring produced) in early life than control females. After the treatment, when circumstances become favourable because of the unlimited access to oviposition substrates, we expected females in the restricted group to increase their reproductive investment relative both to the previous phase (when resources were limited) and to that of control females after the treatment. Second, as investment in reproduction is expected to entail survival costs, it may be expected that females in the control group, by realizing a strong reproductive investment early in life have shorter lifespans than females in the restricted group. However, as costs of reproduction may be contingent on the individual age and/or the environmental conditions previously experienced^14,15^, it can be also expected that females in the restricted group, by experiencing adverse environmental conditions and realizing a higher reproductive investment at a more advanced age (when conditions for reproduction improved), would end up having reduced longevity than control females. Third, we also expected that the current/future reproduction trade-off caused by the experimental setup would have consequences for the behaviour of individuals. In particular, females previously experiencing constraints for oviposition would be expected to search intensively for good quality resources. Thus, we expected that females in the restricted group would show higher movement activity after the treatment (but not before the treatment) than control females.

## Materials and methods

### Study system and study population

We used the seed beetle *Callosobruchus maculatus* (Chrysomelidae, Bruchinae). In the laboratory, these beetles are kept under conditions (dry legume storage environments; see below) that mimic the conditions in which they have evolved for thousands of generations, since this species has adapted to exploiting dry seeds in human grain storages for several thousands of years^16,17^. In our study, we used one of the preferred hosts of this beetle, the mung bean (*Vigna radiata,* hereafter referred simply as beans*)*. After mating, the inseminated females glue eggs on the surface of the beans. After hatching, the first larval instar burrows into the bean’s endosperm where it feeds and completes development. Importantly, females are able to discriminate clean from previously infested beans^18^. Whenever possible, females prefer to distribute their eggs uniformly (1 egg/seed), trying to avoid laying eggs on beans on which an egg (own or non-own) has already been deposited. This is because only a very small fraction of eggs deposited in an already parasitized bean develop successfully as a result of bean size limitations and larval competition^19^. When host deprivation is maintained for a long time (> 4 days^20^) females may lay eggs on unsuitable substrates as well. In our population, infested *Vigna radiata* beans typically contain a single larva developing inside, and generally, a bean of this species provides resources to support only the development of one individual^21^. The species is sexually dimorphic, has a short generation time (< 25 days under our lab conditions), and high mating rates with individuals from both sexes reproducing soon after they emerge as adults^22,23^. Adults do not need to feed or drink, thus individuals obtain all resources for reproduction and survival from the seeds they feed upon during the larval stage. All these features make this system a suitable model for experimental studies, including those targeting life-history traits^17,22,24,25^.

The population used for the experiment was established in 2013 in the Estación Biológica de Doñana (Seville, Spain) with over 450 individuals from a culture tracing back to the original natural population in South India^22^. The population in our laboratory is typically maintained across 3–4 replicated population containers. Each generation and for each replicate, 50 individuals (25 males and 25 females) of ca. 2 days-old are randomly selected and transferred into a new container with clean beans ad libitum to generate the next generation of beetles. Mating and oviposition are allowed for 48 h, after which the 50 reproducing adults are removed from the containers and the new generation started. The effective population size for each replicated population container exceeds 75 individuals, due to the use of already mated females and the high rates of female multiple mating in these populations. Usually, around a thousand adult beetles emerge from each of these containers, and individuals from the different replicated populations are mixed every few generations. Thus, we keep the stock population with large population sizes (in excess of 300 individuals) and non-overlapping generations. Beetles from the stock population are all kept in climate chambers at 29 °C, 40% relative humidity, and a 12L: 12D cycle. Under these conditions egg to adult development occurs in 21–24 days. These breeding conditions imply low levels of larval competition.

### Experiment 1: Long-term responses to restricted early reproduction (hereafter: long-term experiment)

The scheme for the experimental setup is given in the Fig. 1. First, to obtain a large number of unmated individuals of similar age for the experiment, we haphazardly collected and individually isolated in 1.5 ml Eppendorf tubes c.a. 800–1000 inoculated beans (with one larva each inside) from the stock population. One day after emergence, we randomly created groups of three unmated females, one focal and two non-focal females, but maintained the individuals in isolation. The non-focal females were marked with a paint dot (Uni Paint PX 21, uni ball, UK) on the elytra, after immobilizing them on dry ice, to allow subsequent identification of focal (non-painted) females in each group. Focal females were also immobilised on ice and manipulated in exactly the same way, but without marking them. A pilot study confirmed that marking does not affect female life history or behaviour.

The following day (day 2) we performed the first behavioural assays for movement activity (detailed below), after which each focal (non-painted) female was immediately transferred to an experimental container (30 ml plastic containers) together with two non-focal (painted) females and three unmated males, thus mimicking a situation in nature wherein females interact with other individuals (competitively with other females and reproductively with males). All six individuals within each container were of the same age. We randomly split the samples and assigned them into two treatments (see sample sizes in Table 1). Half of the containers were supplied with 120 beans (40 beans/female), in a mixture of 105 already-used and 15 unused beans (“restricted” treatment). Used beans had been exploited for reproduction prior to this experiment by other females; they were beans upon which larvae had fed and developed into adults precluding the development of additional individuals. Therefore, in the restricted treatment (5 unused bean/female), females were exposed to intense competition for suitable oviposition substrates. This is a frequent scenario in nature for the species because, after colonizing a new source of grain, rapid population growth leads to high population densities and severe resource constraints^23^. The other half of the containers included the same number of beans (*N* = 120), but each of these was unused, resulting in plentiful opportunities for oviposition and a relaxed competition among females (“control” treatment). In this phase (treatment phase, hereafter), individuals within each container were allowed to mate and lay eggs for 2 days (until day 4).

**Table 1:** The number of focal females that have been used in the different treatments and blocks of the experiments.

	Control treatment	Restricted treatment	Total
**Long-term experiment**	104	104	208
Block 1	33	33	66
Block 2	10	11	21
Block 3	38	37	75
Block 4	23	23	46
**Short-term experiment**	30	30	60

The final sample sizes available for different analyses (indicated for each specific analysis in Tables 2, 3, 4) may slightly vary from these reference numbers, as a few individuals escaped or died in different phases of the experiment (i.e. during the behavioural assays or in the 1-day oviposition vial). In each analysis, we used the maximum number of available observations. Note that behavioural assays (before and after the treatment) were conducted only on focal females from the long-term experiment.

On day 4, we conducted another round of behavioural assays on the focal females, and subsequently they were individually placed in oviposition vials (30 ml plastic containers) for 1 day (“1-day vial” hereafter). 1-day vials were unanimously supplied with unlimited resources (*N* = 85 unused beans), thus both groups faced the same relaxed conditions for oviposition after the treatment period. We counted the number of eggs (fecundity) deposited on the substrate within these vials and the number of adults emerging from them (reproductive success) to estimate the productivity of isolated females immediately after exposure to the two experimental manipulations. Eggs (hatched or unhatched) are clearly visible on the surface of beans at all times and we counted them 11 days after females were removed to avoid any potential impact on first instars larvae^26^.

On the next day (day 5), focal females were individually transferred to new oviposition vials with ad libitum resources, and kept there for a week (“1-week vial” hereafter). In the 1-week vials, we also counted the number of eggs and the number of adults emerging from them. Vials were checked daily to record longevity. No female survived beyond the 1-week oviposition vial and most individuals died while in this vial, whereas in very rare cases mortality occurred in the 1-day vial.

Movement activity was measured both before and after the treatment (see Fig. 1). To assess movement activity, we placed individuals into separate glass tubes (3.8 cm high, 1 cm diameter). *C. maculatus* beetles typically move up then fall down along the wall of their home containers, a behaviour that, as shown in a pilot study, exhibits a considerable repeatability in the short-term (across different 10-min batches within 1-h recording: N = 39, R = 0.62 (95% CI 0.42/0.74) and long-term (across 3 days of difference: N = 39, R = 0.43 (95% CI 0.13/0.66). Previous evidence also shows that similarly assessed movement is a good proxy of other important functional traits (such as metabolic rate and mating rate) in *Callosobruchus* species^11,12^. Glass tubes were aligned vertically in front of a high-resolution camera. The camera that recorded the traces of movements was connected to the software Ethovision 12XT (Noldus Information Technology, The Netherlands), which provided the absolute length (in millimetres) of movement. Movement activity was recorded for 65 min. We discarded the first 5 min of recordings to negate residual effects of handling and the presence of the experimenter in the vicinity. We could simultaneously record 12 individuals that were arranged along a 2 × 6 (rows by columns) design, and the position of the test tubes were considered in the statistical analyses (tube ID). Within the 2 × 6 design, individuals were randomly positioned with respect to the treatment and beetles were always introduced and removed from the tubes in the same order (from position 1 to 12). As a control, we always added one tube containing a dead animal to calibrate the minimum of movement distance between two consecutive frame rates, assuring that immobile individuals are truly recorded with zero distance. To avoid overestimation of distance due to incidences of falling downs along the tubes (that are not considered as true walking distances), we also set the maximum distance moved between two video frames at 1 mm. This maximum value (1 mm) was estimated by using a subset of individuals, in which we recorded the maximum distance moved between two consecutive frames as well as the distance moved during the falls. As the latter was greater than the former, we could set a threshold to reliably exclude falls from the raw measurements. Behavioural assays were conducted between 10:00 a.m. and 14:00 p.m., and time was included in the models to account for potential fluctuation in activity within the day. Assays were conducted at similar conditions of temperature and humidity than those used to maintain the stock population.

Body size, estimated from elytron length^26^, was measured on focal females at the end of the experiment and included in the statistical models to account for potential differences in activity or life history due to this trait^27^. Images of the females were taken using a stereomicroscope SteREO Discovery.V8 connected to a camera AxioCam Icc 1 (Carl Zeiss, Germany), and the right elytron was measured using the software ZEN 2 (blue edition, Carl Zeiss, 2011). Elytron length exhibits very high (ranging from 0.92 to 0.99) and significant repeatabilities in several repeated samples of over hundreds of individuals from previous assays (Rodriguez-Exposito and Garcia-Gonzalez, unpublished). Body size was also measured blindly with respect to treatment allocation.

To provide independent replications, the above experimental procedures were performed 4 times (experimental blocks, hereafter; see sample sizes per treatment for each experimental blocks in Table 1). We did not use data on longevity from the first experimental block given that we had not checked mortality with the required frequency (i.e., daily) to accurately determine the longevity of individuals. Furthermore, in the first experimental block, some individuals (n = 38) spent three days (instead of two) in the experimental containers. As a result, for these individuals, the steps performed after the treatment phase (behavioural assays and placement in 1-day and 1-week vials) were delayed one day relative to the scheme on Fig. 1. To control for this potential confounding effect, we included the number of days spent in the experimental containers and the age of individuals in the statistical models (results remained qualitatively unchanged if data from the first experimental block were excluded). Focal life-history traits were measured blindly with respect to the experimental treatment and behavioural data.

### Experiment 2: Quantifying current and future reproduction (hereafter: short-term experiment)

Due to the large number of manipulations and recordings, we were unable to measure reproductive investment during the treatment phase (i.e. during the first reproductive round) of the long-term experiment. Therefore, we performed another experiment wherein, in essence, we repeated the long-term experiment until the end of the 1-day oviposition vial, but without performing behavioural assays, to estimate reproductive investment during the treatment phase. The other simplification was that females were not painted, thus we randomly selected one female from each container to be transferred into the oviposition vial for measurements of post-treatment reproductive output. Further, in the containers used in the restricted treatment, we carefully “cleaned” the used beans (those previously used for reproduction and, therefore, useless for the development of additional individuals) by scratching one by one the old eggs and eggshells from them to make sure that these eggs did not confound our estimates of oviposition rates in the experimental treatment containers. The other conditions (i.e. number of used/unused beans, number and age of individuals/sexes per container) were the same as in the long-term experiment. The sample sizes were 30 containers per each of the two groups, restricted and control (of which we lost 1 individual before the completion of protocol; Table 1).

In the short-term experiment, we estimated average *per capita* fecundity during the treatment phase by dividing the number of eggs in each container by three. Similarly, we also counted the number of adults that emerged from these eggs to estimate the average *per capita* productivity during the treatment phase. In the 1-day oviposition vials, we counted the number of eggs and adults emerged from them. To allow for a direct comparison of fecundity/productivity between experimental phases (during vs. after the treatment) with different lengths (2 days vs. 1 day), we divided per capita fecundity and productivity during the treatment phase by two (days) to obtain reproductive estimates per capita and day. Body size was measured as explained above, and all data was taken blindly with respect to experimental treatment.

### Statistical analyses

Before interpreting the statistical results, we systematically performed data exploration and model diagnostics statistics to avoid misleading results based on statistical artefacts (e.g. by checking distributions of raw data, model residuals, multicollinearity and the effect of influential data points^28,29^). Overall, exploratory analyses showed that the number of eggs and number of adult emerged from those eggs were strongly correlated within experimental groups, across the two phases of both experiments (all r > 0.95; p < 0.001). However, there was as an exception to this general correlation since, during the treatment phase of the short-term experiment, females under restricted conditions had a low rate of egg-to-adult viability (16.6% compared with the 83.89% after the treatment), so the relationship between the two reproductive measurements for this group in this phase was not significant (r = 0.05; p = 0.76). Due to the overall high correlation between both life history traits, the number of adult offspring was not considered in further analyses (but see discussion). Further, based on model diagnostics, movement distance was square-root transformed. After that transformation, the diagnostic analyses on the final models showed no obvious deviations from the assumptions of linear models (e.g. there were not problems of collinearity nor influential data points).

#### Short-term experiment

We used Linear Mixed Models (LMM) to investigate the effects of treatment on fecundity. In that model, we included body size, treatment, the underlying experimental phase (during vs. after the treatment) and the interaction between treatment and phase as predictors, whereas female identity (there were two observations for each individual) was included as random factor. Further, by sorting the data to have one row for each individual, we used a linear model (LM) to investigate the relationship between fecundity during and fecundity after the treatment. This model included fecundity after treatment as the response variable, while body size, treatment, fecundity during the treatment, and the interaction between these two latter variables were included as fixed effects.

#### Long-term experiment

To test whether the experimental treatment affected subsequent life history decisions, we fit separated LMMs for the fecundity and longevity variables covering the post-treatment life of the individuals (note that reproductive variables were not recorded during the treatment in the long-term experiment). In the fecundity model, we entered the number of eggs as the response variable while including treatment, experimental phase (1-day and 1-week vial) and post-treatment activity (as energetic investment in activity might affect investment in reproduction) as well as their two-way interactions, and the three-way interaction as fixed effects. The three-way interaction was included to control for potential effects of activity on fecundity that vary across treatments and experimental phases. Further, body size, days in the treatment and age at entering the 1-day vial were also included as control predictors (their interactions were not of interest under the given hypothetical framework). Experimental block, female identity and the position of the recording tube during the behavioural assays were entered as random effects. The longevity model contained treatment, the total number of eggs after the treatment (to assess the treatment effects on longevity independent of fecundity) and post-treatment activity (as energetic investment in activity might reduce longevity) as main terms as well as the two-way interactions between treatment and fecundity, and between treatment and post-treatment activity (“experimental phase” is not relevant in this model as longevity is summed over the phases).We also included body size and age at entering the 1-day vial as fixed effects, while experimental block and the position of the recording tube during the behavioural assays were included as random effects. As data for longevity were unreliable in the first experimental block, the corresponding model relied on data from blocks 2–4 only (in which females spent exactly 2 days in the treatment containers), thus the control for the number of days in the treatment was not applicable.

Before investigating determinants of movement activity after the treatment, we first checked if movement activity assessed before the treatment did not differ by chance between the restricted and control groups. For this, we fitted a LMM including movement activity before the experimental treatment as the response variable, treatment, time at the moment of assay and body size as fixed predictors, and experimental block and the position of the test tube as random factors. This model yielded no differences between the two experimental groups in movement activity before applying the treatment (Likelihood Ratio Test, LRT: χ^2^ = 0.282, *P* = 0.596). Therefore, we continued by fitting a LMM to investigate the determinants of movement activity assessed right after the treatment. In this model, treatment, pre-treatment activity, the interaction between treatment and pre-treatment activity, age at the behavioural assay, time of the day when the assay was done (10.00 to 14.00 h), and body size were included as fixed predictors, while experimental block and position of the recording tube were the random factors. We also checked for consistent within-individual change (increase or decrease) in behaviour before and after treatment with a paired t-test that used the respective behavioural data for each individual.

Statistical analyses were performed in R.3.6.2^30^. For the mixed modelling, we used the package *lme4*^31^*.* Significance of the fixed effects in the models was calculated with Type II (Type III in the presence of significant interactions) Wald Chi-Square tests, using the function Anova (*car* package^32^) on maximum likelihood models, while parameter estimates were calculated using Restricted Maximum Likelihood^33^. The package *HLMdiag*^28^ and the VIF function (*car* package) were used for model diagnostics.

## Results

### Short-term experiment

**Figure 2:**
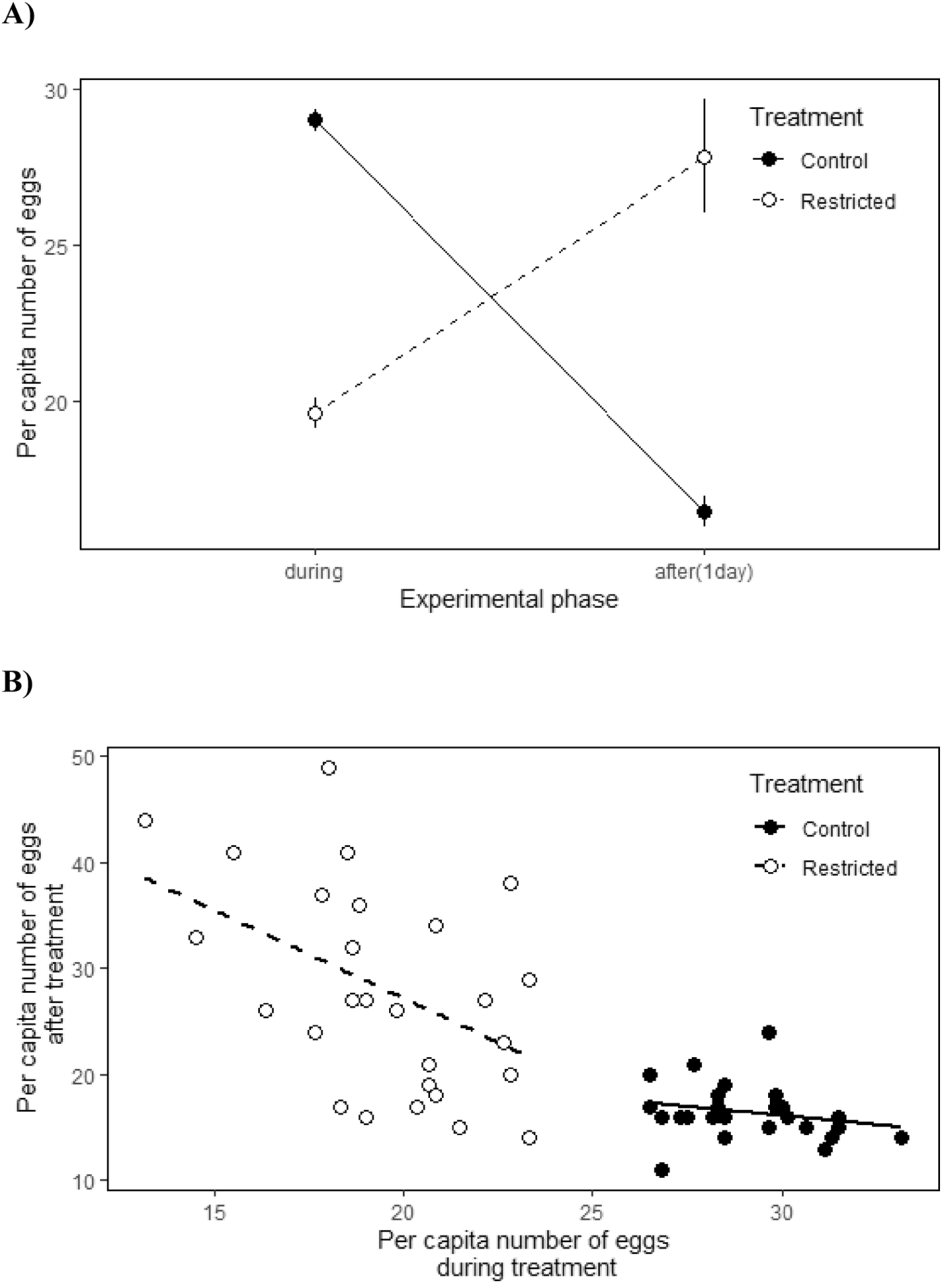
Short-term experiment: relationship between resource availability and reproductive output for the two experimental groups (restricted and control). (**A**) Number of eggs during and one day after the treatment (error bars reflect standard errors around the mean). (**B**) Relationship between current (during treatment period) and future (after treatment period) reproductive investment. Note that this experiment finished one day after the treatment and that we estimated average per capita reproductive output per container and day (see main text).

The effect of treatment on fecundity varied over the two phases of the experiment (interaction treatment*phase; Table 2). Females under the restricted treatment laid fewer eggs than control females during the treatment period, while the opposite was true after the treatment (when individuals from both groups were placed under unlimited number of beans), as females that had previously experienced resource restrictions realized higher fecundity than those in the control group (Fig. 2A). When we assessed the trade-off between current and future reproductive investment, we found that fecundity after the treatment was significantly and negatively related to fecundity during the treatment in the restricted group, but not in the control group (LM: restricted group slope: *β* ± s.e. = − 0.831 ± 0.304, *t*_27_ = − 2.735, *P* = 0.011; control group slope: *β* ± s.e. = − 0.172 ± 0.140, *t*_24_ = − 1.221, *P* = 0.233; interaction treatment * fecundity during treatment: *β* ± s.e. = 0.74 ± 0.41, *t*_27_ = 1.78, *P* = 0.08; Fig. 2B). Overall, summing the two experimental phases, control females showed higher fecundity (mean ± s.e., during: 58.03 ± 0.91; after: 16.46 ± 1.39) than females under restricted conditions (during: 39.22 ± 0.88; after: 27.85 ± 1.34; *t*_56_ = − 2.611, *P* = 0.012).

**Table 2:** Short-term experiment: results of a linear mixed model testing the treatment effects of limiting resources on fecundity (during and after the treatment).

Random effects	σ2	SD				
Female ID	0	0				
Residual	27.91	5.283				

Fixed effects	β	SE	df	Wald X^2^	Wald test df	P-value
Intercept	10.664	16.561	103			
Treatment [restricted]	− 9.460	1.44	103	0.894	1	0.344
Phase [after]	− 12.558	1.465	103	3.184	1	0.074
Body size	8.733	7.842	103	1.3	1	0.254
Treatment [restricted] * phase [after]	20.802	2.035	103	109.595	1	< 0.001

Parameter estimates and SEs were calculates using REML models. *N*_Control_ = 26; *N*_Restricted_ = 28.

### Long-term experiment

#### Behaviour

**Figure 3:**
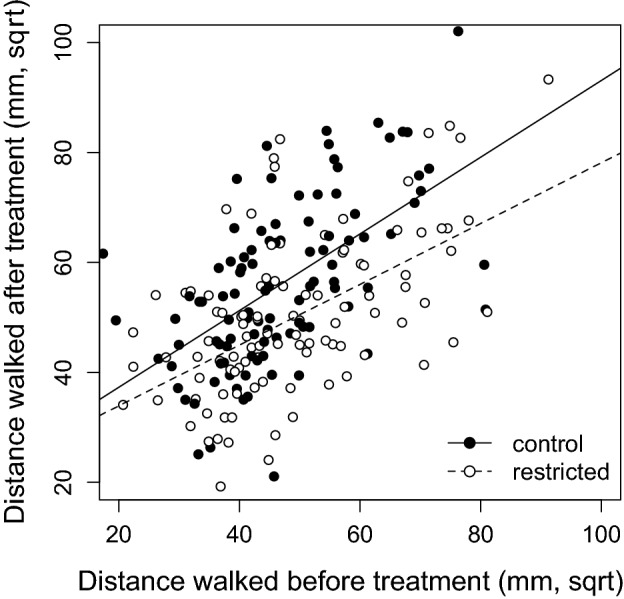
Long-term experiment: movement activity (after square-root transformation) assessed before and after the experimental treatment in the two experimental groups (restricted and control).

The group of females exposed to restricted conditions for egg laying showed lower movement activity after the treatment (mean ± s.e.: 2430 ± 159.7 mm) than the control group (3176 ± 180.9 mm; Table 3; Fig. 3). The within-individual correlation of movement activity across the two assays was considerable in both groups (control: *r* = 0.577, *N* = 100, *P* < 0.001; restricted: *r* = 0.545, *N* = 102, *P* < 0.001) indicating that the order of individuals along the level of trait expression is highly consistent even 2–3 days apart. Thus, as shown by the pairwise patterns, the observed difference in the activity levels between groups after the treatment was caused because females in the control group increased their activity post-treatment relative to that exhibited early in life (*t*_99_ = 7.091, *P* < 0.001), whereas females from the restricted group showed similar movement activity before and after the treatment (*t*_101_ = 0.725, *P* = 0.470; Fig. 3).

**Table 3:** Long-term experiment: result of a linear mixed model testing the treatment effects of resources limitation on activity measured after the treatment.

Random effects	σ2	SD				
Tube ID	0	0				
Exp. block	4.16	2.04				
Residual	137.28	11.72				

Fixed effects	β	SE	df	Wald X^2^	Wald test df	P-value
Intercept	11.914	31.578	96.133			
Mov. distance pre-treatment	0.654	0.099	188.531	73.35	1	< 0.001
Treatment [restricted]	0.916	6.386	188.809	18.52	1	< 0.001
Time	0.945	0.923	110.308	1.412	1	0.235
Days in treatment	11.251	2.779	24.239	15.187	1	< 0.001
Age at measurement	− 5.63	2.064	28.998	7.564	1	< 0.001
Body size	1.854	11.366	188.353	0	1	0.996
Mov. distance pre-treatment * treatment [restricted]	− 0.166	0.129	188.665	1.3665	1	0.242

Parameter estimates and SE were calculates using REML models. *N*_Control_ = 95; *N*_Restricted_ = 102.

#### Life history

**Figure 4:**
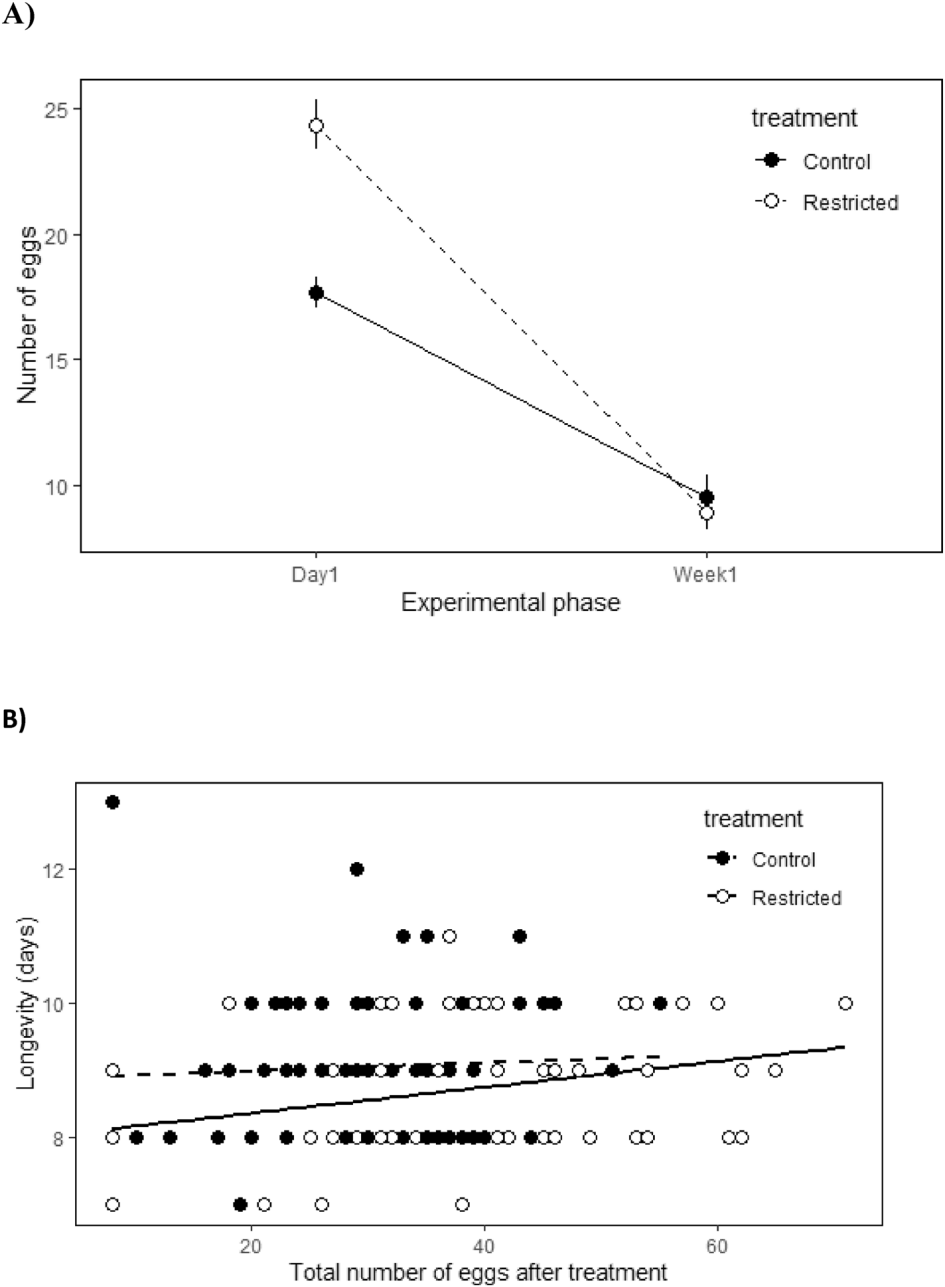
Long-term experiment: relationship between resource availability and reproductive output after the treatment for the two experimental groups (restricted and control). (**A**) Number of eggs laid on the day after the treatment (1-day vial) and the subsequent days (1-week vial). Error bars reflect standard errors around the mean. (**B**) Relationship between the total number of eggs produced after the treatment and longevity. Note that the reproductive investment during the treatment was not estimated in this experiment (see main text).

As occurred in the short-term experiment, the experimental treatment had a strong effect on fecundity immediately after the treatment (on the 1-day vial) as females in the restricted group had higher reproductive investment relative to the control group in that vial. By contrast, the fecundity recorded later on in life, in the 1-week vials, was similar in the two groups (interaction treatment*experimental phase: Table 4, Fig. 4A). Overall, neither activity nor its interactions (two level and the three-way interactions with treatment and/or experimental phase) had an effect on the fecundity after the treatment.

**Table 4:** Long-term experiment: results of linear mixed models testing the treatment effects of limiting resources in early life on subsequent life history decisions: number of eggs (on 1-day vial and 1-week vial; *N*_Control_ = 95, *N*_Restricted_ = 102) and longevity (*N*_Control_ = 67, *N*_Restricted_ = 69).

Number of eggs						
Random effects	σ2	SD				
Female ID	12.351	3.514				
Exp. block	10.526	3.244				
Tube ID	0.432	0.657				
Residual	24.386	4.938				

Fixed effects	β	SE	df	Wald X^2^	Wald test df	P-value
Intercept	− 26.748	12.120	170.191			
Treatment [restricted]	11.612	3.267	307.137	22.160	1	< 0.001
Vial [1-week]	− 12.035	3.032	166.066	549.524	1	< 0.001
Mov. distance	− 0.006	0.043	299.026	0.891	1	0.345
Days in treatment	− 4.692	1.535	185.008	10.926	1	0.001
Age at 1-day vial	− 2.623	0.882	165.980	9.543	1	0.002
Body size	28.524	4.882	163.672	34.967	1	< 0.001
Treatment [restricted] * vial [1-week]	− 7.366	4.019	162.313	44.079	1	< 0.001
Treatment [restricted] * mov. distance	− 0.093	0.059	307.326	3.388	1	0.066
Vial [1-week] * mov. distance	0.062	0.053	168.408	3.152	1	0.076
Treatment [restricted] * vial [1-week] * mov. distance	0.005	0.073	162.726	0.004	1	0.951

Longevity						
Random effects	σ2	SD				
Exp. block	0.101	0.318				
Tube ID	0	0				
Residual	0.588	0.767				

Fixed effects	β	SE	df	Wald X^2^	Wald test df	P-value
Intercept	3.71	2.069	127.575			
Treatment [restricted]	− 0.115	0.601	126.962	19.23	1	< 0.001
Total number of eggs	0.047	0.011	126.517	33.901	1	< 0.001
Mov. distance	− 0.003	0.007	126.823	0.631	1	0.427
Age at 1-day vial	1.134	0.148	107.562	63.841	1	< 0.001
Body size	− 0.507	0.931	126.952	0.259	1	0.611
Treatment [restricted] * total number of eggs	− 0.011	0.012	126.059	0.87	1	0.351
Treatment [restricted] * mov. distance	− 0.002	0.009	127.043	0.095	1	0.758

Parameter estimates and SE were calculates using REML models.

The experimental treatment also influenced longevity (after taking account the positive effect of fecundity on longevity, Fig. 4B), with females in the restricted treatment having reduced longevity (mean ± s.e: 8.68 ± 0.21 days) relative to control females (9.27 ± 0.21 days; Table 4). The activity post-treatment or its interaction with treatment did not explain longevity (Table 4).

## Discussion

We have shown that manipulation of reproductive resources in early life affected (i) investment in future reproduction, as females exposed to limiting resources had lower oviposition rates during exposure to these conditions, but increased fecundity afterwards in relation to control females; (ii) longevity, as individuals in the restricted group, realizing a heavy post-treatment reproductive investment, experienced shorter longevity than control individuals; and (iii) behaviour, as constraints on the access to laying substrates subsequently precluded them from reaching the activity levels attained by control individuals.

Unlike individuals in the control groups, females under the restricted treatment had a low rate of egg-to-adult viability (16.6%) during the treatment phase compared to that after the treatment (when resources became plentiful: 83.89%), indicating that our treatment effectively exposed individuals to constrained reproductive conditions. Life history theory predicts that when environmental circumstances are suboptimal for reproduction, individuals should primarily invest in self-maintenance to increase their survival prospects until conditions for breeding become favourable^4,34^. Accordingly, we detected a negative relationship between current and future fecundity in females initially exposed to adverse conditions for reproduction. Interestingly, the restricted group showed larger variance in fecundity after exposure to restricted oviposition sites than the control group (Fig. 2A). This may suggest that some individuals are permanently impacted by the treatment and others can rebound. Female seed beetles may plastically adjust egg size^25,35^, but it is largely unlikely that a trade-off between number and size of eggs systematically biased our analyses on the reproductive performance of individuals because (i) the main determinants underlying eggs size variation (whether plastically adjusted or not) were randomized and/or controlled in our experiment (e.g. body size, individual age, existence of multiple mating, density as well as the type and size of seeds^25,35^) and (ii) oviposition decisions based on seed availability (number of unused vs already infested beans) are much more important determinants of female fitness^25,35,36^.

The induced changes in the reproductive trajectories of individuals also had an effect on longevity. It is generally assumed that a heavy investment in early-life reproduction increases reproductive success at the cost of survival prospects^34^. In our experiment, control females had high fecundity early in life, which also translated into an overall higher fecundity over the entire experiment (i.e. the sum of the number of eggs laid during and after the treatment was higher than in the restricted group). However, we found that females from the restricted group, which realized a higher reproductive investment after the treatment (relative both to the previous phase and to that of the control group after the treatment), had a reduced lifespan. These results suggest that a strong reproductive investment involves greater costs with advancing age, possibly because such investment exceeds the optimal levels for these ages. Additionally, it is possible that costs of reproduction are dependent upon the environmental conditions experienced over life^14,15,37^. For instance, intense intra-sexual interactions for oviposition resources, implying increased energy expenditure and/or somatic damage, could have resulted in decreased energy stores and/or rapid deterioration, which would have then increased the costs of subsequent reproduction and reduced longevity in the restricted group through carry-over effects. It is important to note that *C. maculatus* beetles are capital breeders that obtain the resources for reproduction and maintenance from the seeds during larval stage (i.e. adult individuals are aphagous^17^), and our experimental design ensured that the potential among-individual differences in resource acquisition during development were similar in the restricted and control groups. Thus, the correlation of life-history traits detected among individuals reflect true trade-offs, likely due to differences in resource allocation decisions^6,7,38,39^.

After the treatment, females from the restricted treatment exhibited lower movement activity than those from the control group. This difference was caused by the fact that individuals in the control group actually shifted their activity levels upwards relative to an initial state early in life, while movement activity in individuals from the restricted group was similar before and after treatment. By moving larger distances, pest insects increase their likelihood of finding optimal environmental conditions, food resources, shelter, mating partners or egg-laying substrates when these are limited^11,13,40^. Therefore, differential activity between treatments might result from a differential investment strategy, whereby individuals that have achieved a high reproductive output early in life could increase their movement activity to find better quality environments (e.g. with high availability of unused beans and/or low intra-sexual competition) to favour egg-to-adult survival in subsequent reproductions. This possibility would have important repercussions for life history theory. If resource restriction early in life, at the onset of first reproduction, has effects on both life history traits and behaviour, such that the activity levels in individuals not experiencing restrictions allow them to obtain greater gains in the future compared to previously constrained individuals, then fitness landscapes as defined by traditional life history traits (fecundity and longevity) may be underestimated to some extent. Our results highlight the need to focus on the potential carry over effects of life-history trade-offs on other traits (namely behaviour), because these effects may have strong consequences on reproductive decisions or on key dynamics (dispersal, prey–predator, etc.) at different levels (individuals, populations, communities), which may go unnoticed when only classical life history trade-offs are measured. A second possibility that could explain the differences in activity between females in the two experimental groups is that females in the restricted group could had withheld egg-laying to some extent until subsequent breeding opportunities^20,21^ and, therefore, they could have been somehow physiologically constrained due the burden of carrying larger loads of eggs. On this point, it is worth noting that, in the fecundity model, we found a trend for an interaction between activity and treatment on fecundity (*P* = 0.066; Table 4), such that activity was negatively related to fecundity in the restricted group, but positively related to fecundity in the control group; this interaction would fit with the notion that the difference in activity levels between the two experimental groups after the treatment could have been due to movement constraints due to the load of unlaid eggs in restricted females.

In conclusion, by manipulating the breeding conditions early in life, we exposed a trade-off between current and future reproductive investments that was intimately connected with the expression of a behavioural trait. Our findings thus suggest intricate feedback loops between life-history and behavioural traits.

## Data Availability

The videos and data sets compiled for our analyses will be deposited on Dryad upon acceptance.
